# Simultaneous thermal camouflage and radiative cooling for ultrahigh-temperature objects using inversely designed hierarchical metamaterial

**DOI:** 10.1515/nanoph-2024-0193

**Published:** 2024-07-11

**Authors:** Saichao Dang, Wei Yang, Jialei Zhang, Qiwen Zhan, Hong Ye

**Affiliations:** Department of Thermal Science and Energy Engineering, 12652 University of Science and Technology of China, Hefei 230027, People’s Republic of China; Sustainable Photonics Energy Research Laboratory, Material Science Engineering, PSE, King Abdullah University of Science and Technology (KAUST), Thuwal 23955-6900, Saudi Arabia; School of Optical-Electrical and Computer Engineering, University of Shanghai for Science and Technology, Shanghai, 200093, People’s Republic of China

**Keywords:** radiative cooling, thermal camouflage, inverse design, ultrahigh-temperature objects

## Abstract

Sophisticated infrared detection technology, operating through atmospheric transmission windows (usually between 3 and 5 μm and 8–13 μm), can detect an object by capturing its emitted thermal radiation, posing a threat to the survival of targeted objects. As per Wien’s displacement law, the shift of peak wavelength towards shorter wavelengths as blackbody temperature rises, underscores the significance of the 3–5 μm range for ultra-high temperature objects (e.g., at 400 °C), emphasizing the crucial need to control this radiation for the objects’ viability. Additionally, effective heat management is essential for ensuring the consistent operation of these ultrahot entities. In this study, based on a database with high-temperature resist materials, we introduced a material-informatics-based framework aimed at achieving the inverse design of simultaneous thermal camouflage (low emittance in the 3–5 μm range) and radiative cooling (high emittance in the non-atmospheric window 5–8 μm range) tailored for ultrahigh-temperature objects. Utilizing the transfer matrix method to calculate spectral properties and employing the particle swarm optimization algorithm, two optimized multilayer structures with desired spectral characteristics are obtained. The resulted structures demonstrate effective infrared camouflage at temperatures up to 250 °C and 500 °C, achieving reductions of 86.7 % and 63.7 % in the infrared signal, respectively. At equivalent heating power densities applied to the structure and aluminum, structure 1 demonstrates a temperature reduction of 29.4 °C at 0.75 W/cm^2^, while structure 2 attains a temperature reduction of 57.5 °C at 1.50 W/cm^2^ compared to aluminum, showcasing enhanced radiative cooling effects. This approach paves the way for attenuating infrared signals from ultrahigh-temperature objects and effectively managing their thermal conditions.

## 1 Introduction

As electro-optical technology advances, infrared detection devices capable of perceiving infrared radiation have found utility in diverse applications [1]–[3]. Such detectors leverage atmospheric transmission windows, typically ranging between 3 to 5 μm and 8–13 μm, allowing them to effectively capture thermal radiation emitted from the surface of an object [4]–[6]. As per Stephen–Boltzmann’s law, all objects emit radiation according to the equation *E* = *εσT* ^4^ [7]. In simpler terms, the intensity of the mid-infrared (MIR) signal released by an object is directly related to its surface emittance (*ε*) and the fourth power of its absolute temperature (*T*) in mid-infrared band. Consequently, high-temperature power-driven components found in aircrafts and ships, such as converging nozzles of aircrafts (∼680 °C) [8] and micro-turbo jet engine funnels of naval ships (∼400 °C) [9], produce notable thermal signals. Without adequate management of thermal radiation, these transportation facilities can be easily spotted by infrared cameras from surroundings [10]–[17]. Therefore, thermal camouflage is critical for high-temperature objects, particularly those with ultrahigh temperatures.

According to the Wien’s displacement law, the peak wavelengths of the thermal radiation shift to shorter wavelengths as the temperature of blackbodies increases. This implies that the proportion of thermal radiation within 3–5 μm can surpass that within 8–13 μm for ultrahigh-temperature objects, despite the narrower bandwidth. For instance, the emitted thermal radiative power of a blackbody at 450 °C (with a peak wavelength of ∼4 μm) in the 3–5 μm range is 171 % of that in the 8–13 μm range. This underscores the importance of thermal camouflage within the 3–5 μm range for ultrahigh high-temperature objects [18]–[20]. Moreover, high-temperature objects also require effective heat dissipation for safe operation. However, the utilization of an infrared camouflage coating with low emission across the entire infrared spectrum can diminish thermal radiation throughout the infrared band. This reduction hampers the object’s heat dissipation through thermal radiation. Notably, in scenarios where thermal radiation occurs in a non-atmospheric window (5–8 μm) constituting a substantial proportion (e.g., 31 % for a 450 °C blackbody), increasing emissions in this band can lower the object’s temperature and attenuate its infrared signal simultaneously. Hence, this wavelength range can be harnessed for heat dissipation through thermal radiation, effectively cooling a high-temperature object without necessitating additional energy [21–25].

While various wavelength-selective infrared camouflage photonic structures [20–31] have been proposed to effectively cool hot objects with high emission in the 5–8 μm range, many of these solutions, such as lithographic fabrication, prove costly and impractical for practical applications [5], [19]. Additionally, there have been discussions concerning the effectiveness of infrared camouflage and thermal management using multilayer structures [4], [6], [19]–[21] such as ZnS/Ge [4], Si/Ge_2_Sb_2_Te_5_ (GST)/Au [5], Ag/Ge [6], and SiO_2_/Ge/ZnS/Pt/Au [13]. However, due to the instability of applied materials (e.g., ZnS, Ag or GST) at elevated temperatures, these structures are limited to operation below 200 °C. Consequently, there’s a lack of research addressing the effectiveness of infrared camouflage in 3–5 μm and radiative cooling in the 5–8 μm range under ultrahigh temperatures (e.g., exceeding 300 °C). To devise a photonic structure capable of operating at high temperatures while exhibiting these essential optical properties, machine learning introduces a promising pathway for the inverse design process [13], [25], [32]–[34].

In this study, based on a database of high-temperature resist materials, we introduce a novel material-informatics-based framework for the inverse design of multilayer metamaterials. This framework is tailored to achieve low emittance in the 3–5 μm range for ultrahigh-temperature infrared camouflage and high emittance in the 5–8 μm range for effective radiative cooling of these high-temperature objects by emitting thermal radiation into the atmosphere. The inverse design process employs the particle swarm optimization (PSO) algorithm, incorporating spectral properties calculated using the transfer matrix method (TMM), to enable automated refinement of multilayer metamaterial structures through machine learning. To validate the predictions generated by the TMM, we fabricated two multilayer metamaterials as designed and characterized their infrared emittance across the 3–8 μm spectrum under various temperatures (from room temperature to 600 °C). The results not only confirm the accuracy of the TMM predictions but also align with electromagnetic field simulations. The two designed structures effectively achieve infrared camouflage at temperatures up to 250 °C and 500 °C, resulting in significant reductions of infrared signal by 86.7 % and 63.7 %, respectively. At equivalent heating power densities applied to the structure and aluminum (Al), structure 1 demonstrates a temperature reduction of 29.4 °C at 0.75 W/cm^2^, while structure 2 attains a temperature reduction of 57.5 °C at 1.50 W/cm^2^ compared to Al, showcasing enhanced radiative cooling effects, owing to their improved radiative cooling capacities. Furthermore, the material informatics framework we introduce here offers the potential for extending this approach to the design of various complex metamaterials, enabling precise control over multispectral optical properties.

## 2 Materials and methods

### 2.1 Concept design

**Figure 1:**
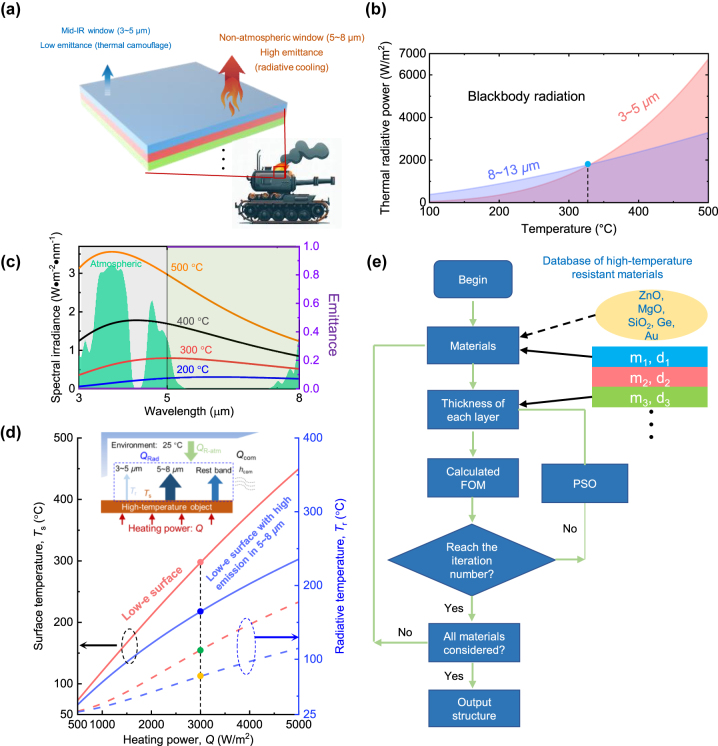
Schematic and optimizing process. (a) Application scenarios for the thermal camouflage in 3–5 μm and radiative cooling in 5–8 μm based on hierarchical metamaterials. (b) The comparison of thermal radiative powers within 3–5 μm and 8–13 μm for blackbodies at different temperatures. (c) The ideal emittance to realize thermal camouflage in 3–5 μm and radiative cooling in 5–8 μm and the spectral irradiance of a blackbody at 200 °C, 300 °C, 400 °C. (d) Surface and radiative temperature reduction caused by high emission in 5–8 μm. Inset shows the energy balance illustration. (e) The schematic of optimization process based on PSO method.

Figure 1(a) illustrates the concept of implementing infrared camouflage combined with thermal management for ultrahigh-temperature objects using multilayer structures. This concept entails achieving low emittance in the 3–5 μm range for thermal camouflage and high emittance in the 5–8 μm range for effective radiative cooling. As depicted in Figure 1(b), the proportion of thermal radiation within 3–5 μm surpass that within 8–13 μm for blackbodies at temperature higher than 331 °C (blue dot), even with the narrower bandwidth. This is due to the significant emission of thermal radiation within the transparent window of 3–5 μm by high-temperature blackbodies, as highlighted in green in Figure 1(c). Low emittance in this range is crucial for successful thermal camouflage. The primary approach for thermal management involves dissipating excess heat through thermal radiation within the non-atmospheric window of 5–8 μm, which is challenging to detect with standard equipment. The ideal spectral characteristics for mid-infrared thermal camouflage with thermal management are represented by the purple lines in Figure 1(c). To assess the effectiveness of temperature reduction through radiative cooling within the 5–8 μm range, we consider two surfaces: one (represented by red lines in Figure 1(d)) with low emittance (i.e., 0.05) across the entire spectrum, and the other (represented by blue lines in Figure 1(d)) with high emissivity (i.e., 1) limited to the 5–8 μm band. The surface temperatures of these two surfaces are determined through an energy balance model as illustrated in the Figure 1(d) inset for comparative analysis:

(1) $$\begin{array}{cc} Q & =\;Q_{\text{Rad}}\left(\varepsilon ,T_{\text{s}}\right)+Q_{\text{com}}\left(h_{\text{com}},T_{\text{amb}},T_{\text{s}}\right)\\ & \;-Q_{\text{R }-\text{ amb}}\left(\varepsilon ,\varepsilon_{\text{amb}},T_{\text{amb}}\right) \end{array}$$

Here *T*_s_ and *T*_amb_ are the surface temperatures of the object and ambient. *Q*_rad_ signifies the thermal radiation emitted by the object (which is proportional to the fourth power of its temperature, multiplied by the Stefan–Boltzmann constant). *Q*_com_ represents the heat loss caused by conductive and convective heat exchange with the ambient, corresponding to *h*_com_ (the combined non-radiative heat transfer coefficient) as illustrated in the inset of Figure 1(d). *Q*_R-amb_ is the absorbed thermal radiation by the object from the ambient (for detailed information, refer to Note S1). As shown in Figure 1(d) (solid lines), when there is high emission in 5–8 μm, the temperature of a low-e surface under a heating power of 3000 W/m^2^ can be reduced by 79.8 °C (as indicated by the red dot: 297.3 °C compared to the blue dot: 217.5 °C). This underscores the significance of radiative cooling within the 5–8 μm spectrum, as it enables the object to operate under cooler conditions. Furthermore, this reduction in temperature contributes to a weakened infrared signal (For the definition of the signal intensity, please refer to Note S2 for details), resulting in a lower radiative temperature (*T*_r_). This radiative temperature, which is detectable by an infrared camera functioning in the 3–5 μm range, can be calculated using the inverse function of *P*(*ε*_i_, *T*_s_):

(2) $$T_{\text{r}}=P^{-1}\left(\varepsilon_{\text{i}},T_{\text{s}}\right)$$

where *ε*_i_ is the default emittance (usually *ε*_i_ = 1) in the infrared camera. *P*(*ε*, *T*_r_) includes the emitted thermal radiation of the object (*P*_rad_) and the ambient radiation reflected by the object (*P*_ref_) with a detailed description, as follows:

(3) $$\begin{array}{cc} P\left(\varepsilon ,T\right) & =P_{\text{rad}}\left(\varepsilon ,T_{\text{s}}\right)+P_{\text{ref}}\left(\varepsilon ,\varepsilon_{\text{amb}},T_{\text{amb}}\right)\\ & =C\overset{5\mu \text{m}}{\underset{3\mu \text{m}}{\int }}\varepsilon \left(\lambda \right)I_{\text{BB}}\left(\lambda ,T_{\text{s}}\right)d\lambda +C\\ & \;\times \overset{5\mu \text{m}}{\underset{3\mu \text{m}}{\int }}\left(1-\varepsilon \left(\lambda \right)\right)\varepsilon_{\text{amb}}I_{\text{BB}}\left(\lambda ,T_{\text{amb}}\right)d\lambda \end{array}$$

where *ε*_amb_ is the spectral emittance and *C* is the angle integral constant (see more details in Note S3). The radiative temperatures, as indicated by the dashed lines in Figure 1(d), clearly demonstrate the impact of emissivity. With both surfaces exhibiting an emittance of only 0.05 within the 3–5 μm range, the radiative temperatures are significantly lower than the respective surface temperatures (dashed lines compared to solid lines). This underscores the ability of low emissivity within the thermal detection band to substantially reduce the signal. For instance, under a heating power of 3000 W/m^2^, a surface with low emissivity and a surface with low emissivity but high emission within the 5–8 μm range are 180 °C and 141.3 °C cooler than their corresponding surface temperatures (indicated by the red dot: 297.3 °C versus the green dot: 112.3 °C on the right *y*-axis, and the blue dot: 217.5 °C versus the yellow dot: 76.8 °C on the right *y*-axis), respectively. Furthermore, it’s worth noting that by incorporating high emission within the 5–8 μm range, the radiative temperature of a low emissivity surface under a heating power of 3000 W/m^2^ can be reduced by 35.5 °C (green dot: 112.3 °C versus yellow dot: 76.8 °C). This highlights the importance of utilizing radiative cooling in the 5–8 μm range for achieving lower operating temperatures and weakening the infrared signal. In this study, we’ve introduced an inverse design approach for creating multilayer metamaterials that achieve thermal camouflage and radiative cooling for ultrahigh-temperature objects. By carefully adjusting the thickness and material composition of each layer, this multilayer structure can be optimized to attain the desired low emissivity within the 3–5 μm range and high emissivity within the 5–8 μm range.

Particle swarm optimization (PSO) is a widely employed technique for designing selective emitters [35]. Leveraging PSO, the inverse design of multilayer structures holds the potential to explore numerous material combinations, utilize thin film layers efficiently, and yield improved spectral characteristics aligned with the evaluation criteria. In this endeavor, a database encompasses five high-temperature resist materials, including four transparent materials within the infrared range (SiO_2_, Ge, ZnO, and MgO), as well as gold metal. The inverse design process is achieved using the PSO algorithm, which considers the infrared spectral properties within the 3–8 μm to identify multilayer structures with exceptional performance [13], [25]. Notably, the figure of merit (FOM) serves as the defining parameter, and it is formulated as follows:

(4) $$\text{FOM}=0.5\left(1-\varepsilon_{3-5}\right)+0.5\varepsilon_{5-8}$$

Here, *ε*_3–5_ and *ε*_5–8_ represent for the average emittance of 3–5 μm and 5–8 μm, respectively. A higher FOM value signifies a more suitable design meeting the requirements. During the inverse design process, we considered 3-layer and 4-layer thin film structures with the substrate of SiO_2_ (500 μm). Each of the inverse-design layers are the thickness between 0 and 1,000 nm. The details of PSO are shown in Figure 1(d). In our utilization of the memetic evolutionary algorithm, the permutation of three or four layers with varying thickness for each layer is engaged in. During this process, the transfer-matrix method (TMM) is employed to determine the spectral properties, as explained in more detail in Note S4. This permutation process continues until it reaches a designated iteration number, at which point it yields the optimized outcome for the current permutation. All possible permutations of three/four layers are scanned based on the seven materials. From the output results of all permutations, we select the best one as the final inverse design. Further discussion and specific settings on PSO can be found in Note S5. The initial swarm is composed of 1,000 particles, which are with randomly thickness (0–1,000 nm) for each layer in the three/four-layer structure. In the PSO, the number of iterations is set as 200 because the FOM would not change for larger values.

### 2.2 Designed results

**Figure 2:**
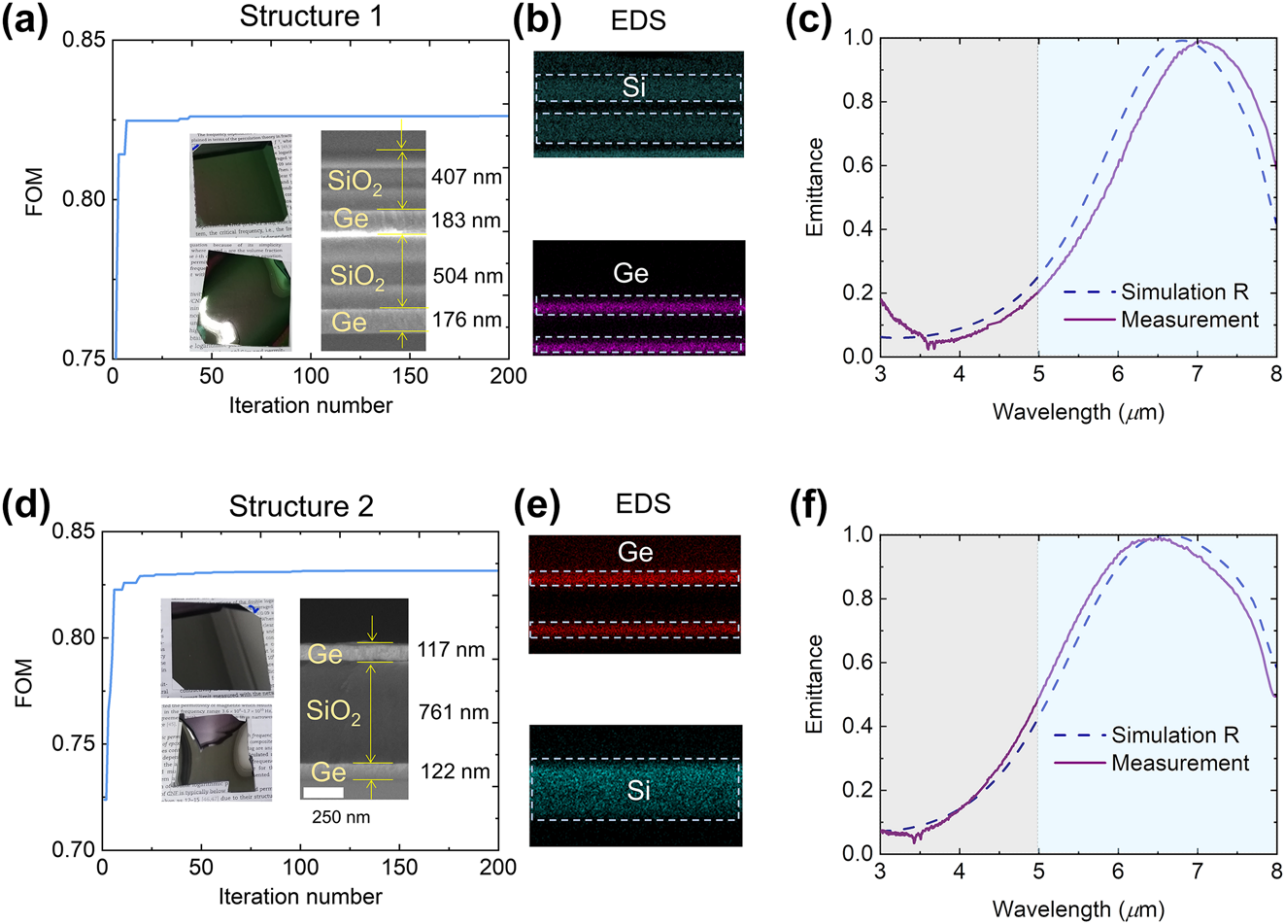
The optimized two structures and the fabricated structure. (a) The optimizing process of the FOM with a function of iteration number. Inset are the prepared samples with SiO_2_ substrate and soft PET substrate, as well as the SEM result. (b) The EDS results of structure 1 to prove the existence of element Ge and Si in different layers. (c) The measured and simulated emittance of structure 1. (d) The optimizing process of the FOM with a function of iteration number. Inset are the prepared samples with SiO_2_ substrate and soft PET substrate, as well as the SEM result. (e) The EDS results of structure 2 to prove the existence of element Ge and Si in different layers. (f) The measured and simulated emittance of structure 2.

By employing the TMM methods and PSO within the Matlab software, the design process takes approximately 1.5 h to yield a single optimized structure on a computer equipped with an 8-core central processing unit (Intel Core i9-11900K). After the entire optimization process is completed, two inverse-design structures are obtained based on the procedure illustrated in Figure 1(e). The FOM’s variation with respect to the number of iterations for structure 1 is depicted in Figure 2(a). As the iteration number increases, the FOM value rises rapidly and then stabilizes, indicating a successful inverse design process. Subsequently, the corresponding structure is fabricated through e-beam deposition (details in Materials and Methods) on a silica substrate (upper panel) or a flexible PET substrate (lower panel), as shown by two photos in Figure 2(a). As depicted in the SEM result in the inset of Figure 2(a), this design yields a multilayer structure consisting of SiO_2_/Ge/SiO_2_/Ge. The energy-dispersive x-ray spectroscopy (EDS) results display the elemental distribution of Ge and Si. In Figure 2(c), the simulated emittance within the 3–8 μm range is presented, along with the measured emittance, which closely aligns with the simulation results.

Furthermore, another three-layer structure is obtained with its corresponding optimization process, as shown in Figure 2(d). This structure, composed of Ge/SiO_2_/Ge (SEM in Figure 2(d)), is similarly confirmed by EDS results displayed in Figure 2(e). The simulated and measured results for this structure are shown in Figure 2(f), and both sets of results exhibit strong agreement. With distinct structures, similar results are obtained. The emittance of the two structures are derived from reflectance and transmittance (i.e., 0) by 1 − *R* − *T* (see details in materials and method). As shown in Figure 2(c)–(f), the measured emittance in 3–8 μm agree well with that of the simulated result. Therefore, our simulation is reliable and the fabricated structures meet the requirement. The measured results demonstrated the high performance of thermal camouflage (structure 1: *ε*_3–5 μm_ = 0.13, structure 2: *ε*_3–5 μm_ = 0.16) and thermal management (structure 1: *ε*_5–8 μm_ = 0.72, structure 2 *ε*_5–8 μm_ = 0.75). According to the Eq. (1), the proposed structures have the high compatible efficiency (structure 1: FOM = 0.80, structure 2: FOM = 0.80). Subsequently, the efficacy of both structures in terms of infrared camouflage and radiative cooling is examined.

### 2.3 Spectral radiative properties

**Figure 3:**
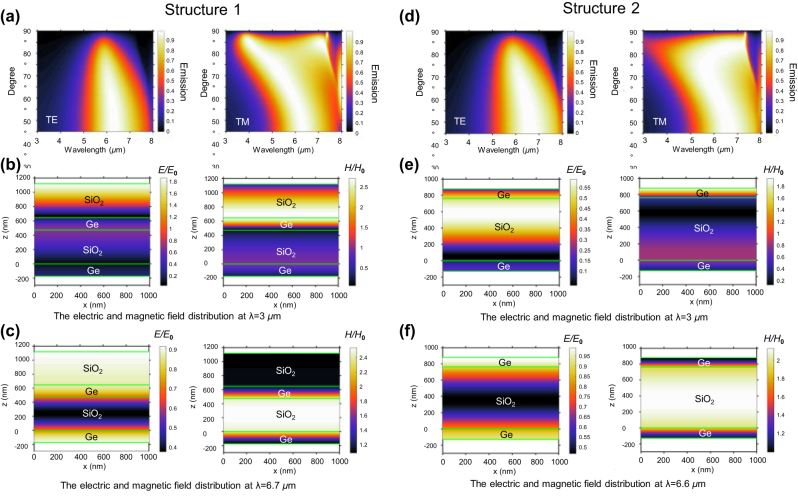
Optical properties of the two structures and the electric and magnetic distribution at *λ* = 3 μm and *λ* = 6.6 μm. (a) The emittance of structure 1 at different incident angles. (b) The electric and magnetic field distribution of structure 1 at *λ* = 3 μm. (c) The electric and magnetic field distribution of structure 1 at *λ* = 6.7 μm. (d) The emittance of structure 2 at different incident angles. (e) The electric and magnetic field distribution of structure 2 at *λ* = 3 μm. (f) The electric and magnetic field distribution of structure 2 at *λ* = 6.7 μm.

Using the optical parameters of SiO_2_ and Ge, we conducted simulations to analyze the detailed optical properties of both structures, aiming for a comprehensive understanding. In Figure 3(a), it’s evident that structure 1, consisting of SiO_2_/Ge/SiO_2_/Ge, remains free from iridescence even at incident angles up to 30° (where 0° denotes normal incident angle). Both TE-polarized and TM-polarized light waves support significant angular incidences up to 30° due to the high refractive index of Ge [13]. For TE waves, at larger oblique incident angles than 30°, the high-emittance band within 5–8 μm becomes narrower, indicating a weaker radiative cooling performance, while the low-emittance band within 3–5 μm becomes wider, indicating an improved thermal camouflage performance. Conversely, for TM waves, the emission peak caused by the resonance effect shifts to shorter wavelengths under larger incident angles, leading to a narrower low-emittance band within 3–5 μm and a wider high-emittance band within 5–8 μm, indicating weaker thermal camouflage performance and a better radiative cooling effect. Analysis of the electric and magnetic field distribution indicates that the radiative properties are a result of interference effects [35–39]. For instance, at *λ* = 3 μm, double electric fields and zero magnetic fields demonstrate the reflection of electromagnetic waves back to the incident direction (top) as shown in Figure 3(b). Conversely, at *λ* = 6.7 μm, the incident electromagnetic wave is absorbed by the structure (Figure 3(c)). Similar results that high reflection (Figure 3(d)) occurs within 3–5 μm (e.g. at 3 μm, Figure 3(e)) and high absorption occurs (Figure 3(d)) within 5–8 μm (e.g. at 6.6 μm, Figure 3(f)) are obtained for structure 2, Ge/SiO_2_/Ge. In other words, different structures yield analogous outcomes, underlining the potential of this inverse design approach to achieve the desired thermal radiative properties.

## 3 High-temperature thermal camouflage and radiative cooling performances

**Figure 4:**
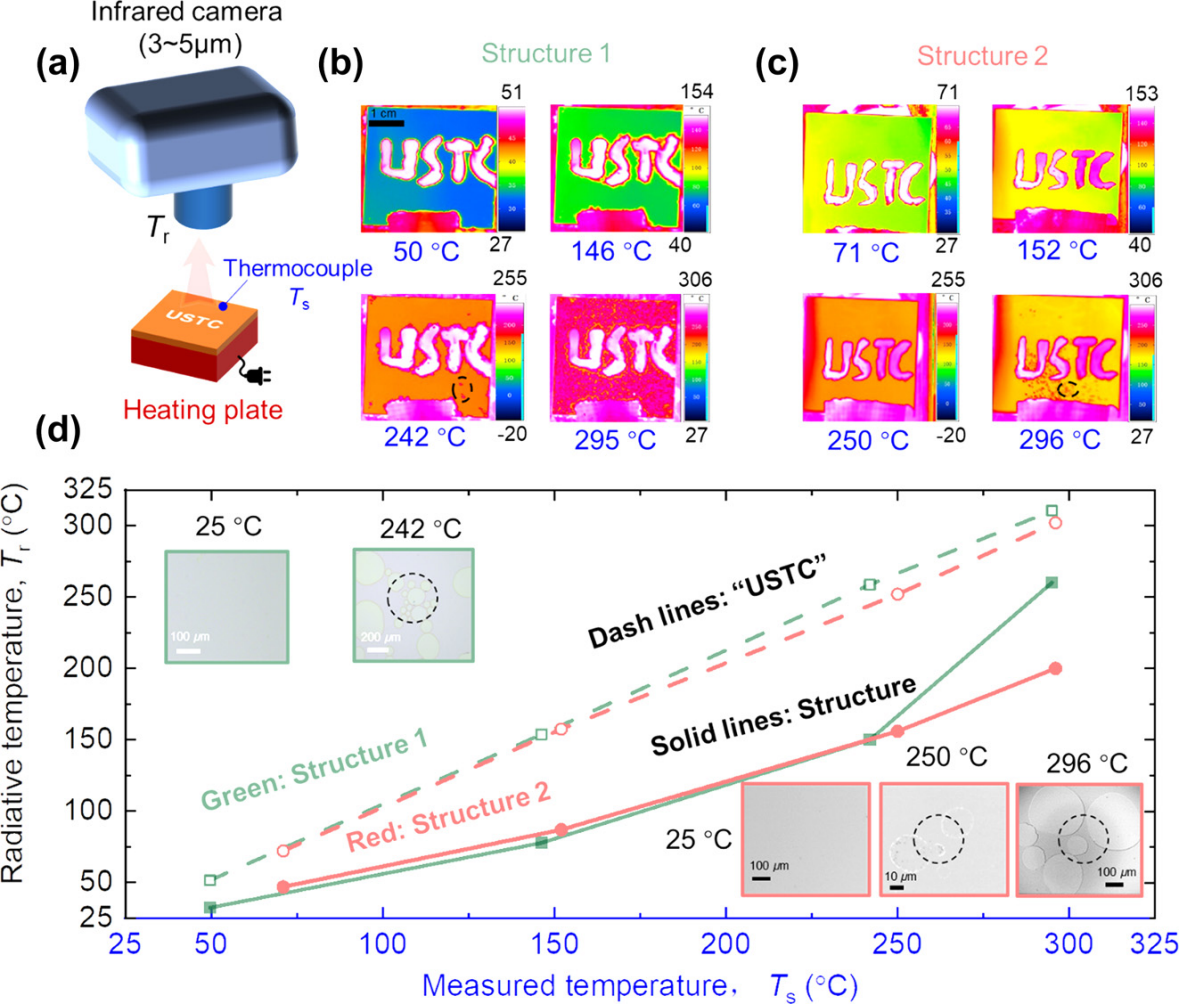
High-temperature camouflage (3–5 μm) performance of the two structures. (a) The schematic for the experiment of high-temperature camouflage. (b) The thermal camera images of structure 1 a under temperatures of 50 °C, 146 °C, 242 °C, and 295 °C, respectively. (c) The thermal camera images of structure 2 a under temperatures of 71 °C, 152 °C, 250 °C, and 296 °C, respectively. (d) The radiative temperature of the structures and high thermal-emissive “USTC” under different measured temperatures from thermal couples. Insets show the surface morphology properties of the structures at different temperatures.

To assess the high-temperature thermal camouflage capabilities of these structures, an infrared camera (FLIR ThermaCAM S65) operating within the 3–5 μm wavelength range is utilized to observe them. The experimental setup involved placing the structures on a heated plate (Figure 4(a)) capable of reaching temperatures up to 600 °C. The actual temperature of the structures is measured using K-type armored high-temperature thermocouples. For comparison, we used thermally emissive adhesive (*ε* = 0.95) to write the letters “USTC” at the center of the structures.

As depicted in Figure 4(b) and (c), both structures reached temperatures of approximately 300 °C, indicated by the blue values. The radiative temperature (*T*_r_) recorded by the thermal camera as a function of the actual temperature is illustrated in Figure 4(d). Structure 1 consistently exhibited a significantly lower radiative temperature at temperatures below 242 °C, and this difference grew as the temperature increased. At 242 °C, structure 1 showcased a reduction in radiative temperature by 119 °C, correlating to a substantial 86.7 % decrease in the infrared signal. However, it’s worth noting that at this temperature, structure 1 began to break, as indicated in the inset plot enclosed by a green line at 242 °C. This damage was also visible in the infrared images shown in Figure 4(b), where dark points circled by dashed lines appeared. When the temperature reached 295 °C, the entire surface of structure 1 fractured, resulting in impaired thermal camouflage.

Upon placing structure 2 on the heated plate, we observed a similar thermal camouflage performance to that of structure 1. However, due to its higher emittance in the 3–5 μm range (0.13 vs 0.16), the radiative temperature of structure 2 was marginally higher than that of structure 1, as depicted in Figure 4(d) at temperatures below 150 °C. Notably, the thermal camouflage performance of structure 2 diverged at temperatures exceeding 250 °C, with signs of structural damage emerging around 300 °C, as evident from the circled region in Figure 4(c). This degradation process was also observed in the inset scanning electron microscope (SEM) images in Figure 4(d). At ∼250 °C, certain regions began to exhibit damage, as circumscribed. By 296 °C, the damaged region was clearly visible. Nonetheless, a significant portion of the structure maintained its integrity at this temperature, and the radiative temperature of structure 2 stayed 100 °C lower than the measured surface temperature, allowing the structure to remain operational in these conditions. In the following paragraphs, we will delve into the thermal camouflage performance of structure 2 at elevated temperatures.

**Figure 5:**
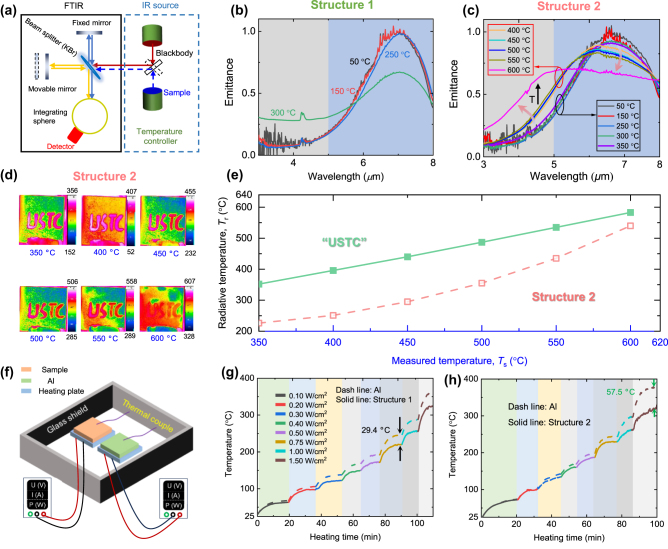
High-temperature emittance of two structures. (a) The schematic to measure the emittance of a sample under different temperatures. (b) The measured emittance of two structures under different temperatures. (c) The measured emittance of two structures under different temperatures. (d) The thermal images of structure 2 under temperatures higher than 350 °C. The infrared images taken by 3–5 μm infrared cameras at different surface temperatures. (e) The radiative temperature of the structures and high thermal-emissive “USTC” under different measured temperatures (>300 °C) from thermal couples. (f) Schematic of thermal management experiment to determine the radiative cooling performance of two structures. (g) The temperature rises with the growing heating power as a function of time for structure 1. (h) The temperature rises with the growing heating power as a function of time for structure 2.

To comprehend the thermal camouflage performance of both structures, their spectral emittance at high temperatures is measure directly using FTIR with an infrared blackbody source, as depicted in Figure 5(a) (see details in Note S6). By comparing the emitted thermal radiation from a blackbody (*S*_B_) and the structure (*S*_S_) at the same controlled temperature, the emittance of the structure can be determined by

(5) $$\varepsilon =\frac{S_{\text{S}}-S_{\text{amb}}}{S_{\text{B}}-S_{\text{amb}}}$$

*S*_amb_ represents ambient thermal radiation. The ambient thermal radiation serves as the background for high-temperature emissivity measurements. At room temperature with high emittance, ambient thermal radiation is comparable to the thermal radiation from the sample and the blackbody at low temperatures. Conversely, at high temperatures, the thermal radiation from the sample and the blackbody significantly exceeds that from the ambient. Consequently, the measured emittance of the sample at a low temperature of 50 °C exhibits strong fluctuations, as shown in Figure 5(b). As illustrated in Figure 5(b), the emittance of structure 1 remains stable at temperatures below 250 °C, characterized by low emittance within 3–5 μm and high emittance within 5–8 μm. At 50 °C, the measured emittance within 3–5 μm is unstable due to the relatively strong ambient signal compared to the blackbody and structure signals. However, as the temperature increases, the signals from the blackbody and structure become stronger, resulting in stable measured emittance. At 300 °C, the emittance of structure 1 increases within 3–5 μm and decreases significantly within 5–8 μm. This temperature coincides with the point of structural failure, aligning with the rise in radiative temperature shown in Figure 4(b).

In Figure 5(c), structure 2 exhibits similar emittance to the results in Figure 2(f) at temperatures below 300 °C. However, at temperatures exceeding 350 °C, the emittance gradually increases within 3–5 μm and decreases within 5–8 μm as the temperature rises. The thermal camouflage performance of structure 2 becomes compromised, as demonstrated in Figure 5(c). At 600 °C, the letters “USTC” almost blend into the structure (Figure 5(d)). Nevertheless, at temperatures below 550 °C, structure 2 achieves a radiative temperature reduction of over 100 °C compared to the high-emittance “USTC” (Figure 5(e)). At a temperature of 500 °C, a radiative temperature reduction of 132 °C is realized, which correlates to a substantial 63.7 % decrease in the infrared signal. It should be noted that the emissive adhesive contains polymer material, which may decompose at extremely high temperatures, causing a slight decrease in the adhesive’s emittance. Consequently, the radiative temperature of the “USTC” is slightly lower than the actual surface temperature when the temperature exceeds 400 °C.

The experiment presented in Figure 5(f) validates the thermal management performance achieved through radiative cooling of the two structures. A control group consisting of identically sized silica covered with an aluminum (Al) film is chosen for comparison. Each sample is heated using a ceramic heating plate, powered by a direct current (DC) power supply. Temperature measurements for each sample are obtained through a thermocouple connected to a data acquisition instrument. To minimize thermal contact resistance, silicon grease is applied between the heating plate and the sample. A glass shield is employed to minimize wind interference. As the heating power increases, the equilibrium temperatures of structure 1 and the Al sample rise, as displayed in Figure 5(g). With identical heating power, the temperature of structure 1 consistently remains lower than that of the Al sample, attributed to its thermal management by heat dissipation through radiation. The temperature gap between structure 1 and the Al sample expands with increasing heating power. At 0.75 W/cm^2^ heating power, the actual temperature of structure 1 (221.2 °C) is 29.4 °C lower than that of the Al sample (250.4 °C). As depicted in Figure 5(h), the cooling performance of structure 2 also improves with rising heating power. With increasing temperature, the contribution of emitted power through the 5–8 μm window likewise increases. At 1.50 W/cm^2^ heating power, the actual temperature of structure 2 (323.9 °C) is 57.5 °C lower than that of the Al sample (381.4 °C). Consequently, both structures exhibit effective cooling performance at high temperatures.

Table 1 presents a comparative analysis between our findings and recent research on infrared camouflage combined with thermal management. The focus of thermal camouflage lies within the 3–5 μm atmospheric transmission window, necessitating low emittance to conceal the hot object within its background environment. Meanwhile, thermal management aims to manipulate thermal radiation across undetectable infrared bands akin to a blackbody. In our assessment, we emphasize the radiative power within the non-atmospheric window (i.e., 5–8 μm) as indicative of thermal management performance. Notably, our structures exhibit advantages in both operating and cooling temperatures. However, while our study emphasizes these specific bands, other research may delve into broader thermal camouflage and radiative cooling spectrums. The prospect of achieving multi-band thermal camouflage and radiative cooling using hierarchical metamaterials tailored for diverse temperature ranges holds considerable promise. Additionally, this scheme may pave the way for advancements in energy-efficient MIR optical materials and devices, while also highlighting the potential for further research into adaptive MIR optical materials and devices [40], [41].

**Table 1:** Comparison of the performance of thermal camouflage and radiative cooling for ultrahigh-temperature objects.

Ref.	Materials	Structure	Thermal camouflage *ε*_3–5 μm_	Thermal management *ε*_5–8 μm_	Working temperature (°C)	Cooling temperature (°C)
[4]	Ge/Al_2_O_3_/Ge/ZnS	Thin films	0.042	0.473	200	14.4
[5]	Si/GST/Au	Gratings	0.25	0.77	×	15
[6]	Ag/Ge	Thin films	0.18	0.82	200	15
[13]	SiO_2_/Ge/ZnS/Pt/Au	Thin films	0.21	0.54	100	7
[14]	ZnO/Ag/ZnO	Gratings	0.39	0.68	180	14.8
[29]	GST/Au	Gratings	0.17	0.85	×	×
[42]	Si/Mo/Si/Mo	Thin films	0.26	0.75	650	×
Structure 1	SiO_2_/Ge/SiO_2_/Ge	Thin films	0.13	0.72	250	29.4
Structure 2	Ge/SiO_2_/Ge	Thin films	0.16	0.75	500	57.5

## 4 Conclusions

Enabling both thermal camouflage and heat dissipation through thermal radiation poses a challenge for ultrahigh-temperature objects for ultrahigh-temperature objects (e.g., >200 °C), given that an object’s thermal radiation is directly proportional to the fourth power of its temperature (*T* ^4^). In response to this challenge, we introduce a comprehensive material-informatics framework for the inverse design of multilayer metamaterials based on a database with high-temperature resist materials. This design aims to achieve low emittance in the 3–5 μm range for thermal camouflage and high emittance in the 5–8 μm range for effective radiative cooling. Our innovative design process merges the Particle Swarm Optimization (PSO) algorithm with the Transfer Matrix Method (TMM), allowing for the automated refinement of multilayer metamaterial structures via machine learning. To validate the accuracy of our TMM-based predictions, we experimentally produce two specified multilayer metamaterial structures and assess their infrared emittance across the 3–8 μm range at varying temperatures (from room temperature to 600 °C). The results reveal that both structures provide effective infrared camouflage between 3 and 5 μm, even at temperatures ranging from 250 °C to 500 °C, leading to substantial reductions of infrared signal by 86.7 % and 63.7 %, respectively. At equivalent heating power densities applied to the structure and aluminum (Al), structure 1 demonstrates a temperature reduction of 29.4 °C at 0.75 W/cm^2^, while structure 2 attains a temperature reduction of 57.5 °C at 1.50 W/cm^2^ compared to Al, due to their enhanced radiative cooling capacities. This innovative approach holds the potential to open up new possibilities in the realm of ultrahigh-temperature thermal management and infrared signal processing. Additionally, our scheme may pave the way for further developments of energy-efficient mid infrared optical materials and devices.

### 4.1 Methods

The multilayer structure is fabricated by e-beam deposition method using e-beam Kurt J. Lesker. LAB 18. The SiO_2_ particles used in the process are from Zhongnuo New Material technology Co. Ltd (China) with a purity of 99.999 % and a diameter between 1 and 3 mm, while the Ge particles come from Fuzhou innovation photoelectric technology Co. Ltd (China) with a purity of 99.999 % and a diameter between 6 and 10 mm. For the deposition of SiO_2_ and Ge, both the growing speeds are 3 Å/s and vacuum pressure of 5 mTorr. The SEM results of the multi-layer structures are obtained through SIRION200.

To measure the direct-hemispherical infrared reflectance of the samples, a FTIR spectrometer (Bruker VERTEX 80) with a detector of DTGS an external golden integrating sphere (A562) was used, and the beam impinges on the sample with an incident angle of 13. The source of the high-temperature blackbody is SiC (see more details in Note S6).

## Supplementary Material

Supplementary Material Details
