# Supplementary material for: Simultaneous thermal camouflage and radiative cooling for ultrahigh-temperature objects using inversely designed hierarchical metamaterial
Source: Nanophotonics. 2024 Jul 11;13(20):3835–46. doi: 10.1515/nanoph-2024-0193 (PMC11466004; doi:10.1515/nanoph-2024-0193)
Supplement: Supplementary file 1 — Supplementary Material Details [file j_nanoph-2024-0193_suppl_001.docx]

Supporting materials for

Simultaneous Thermal Camouflage and Radiative Cooling for Ultrahigh-Temperature Objects Using Inversely Designed Hierarchical Metamaterial

Saichao Dang^1, 2^, Wei Yang^1^, Jialei Zhang^1^, Qiwen Zhan^3^*, Hong Ye^1^*

1. Department of Thermal Science and Energy Engineering, University of Science and Technology of China, Hefei 230027, People’s Republic of China

2. Sustainable Photonics Energy Research Laboratory, Material Science Engineering, PSE, King Abdullah University of Science and Technology (KAUST), Thuwal 23955-6900, Saudi Arabia

3. School of Optical-Electrical and Computer Engineering, University of Shanghai for Science and Technology, Shanghai, 200093, China

* Correspondence:

H. Ye: [hye@ustc.edu.cn](mailto:hye@ustc.edu.cn), Q. Zhan: qwzhan@usst.edu.cn

Note S1 The surface temperature calculation

To predict the improved performances of thermal management and infrared camouflage of a surface structure with a high emission in 5~8 *μ*m, the spectral emittances are presupposed with typical values, as shown in Table s1. Three bands, i.e., 3~5 *μ*m, 5~8 *μ*m and the rest band are taken into account to evaluate the thermal radiation. Both surfaces have low emittance in the rest band. Structure 1 has a broadband low emittance (i.e., 0.05) within 3~8 μm, while structure 2 has low emittance (i.e., 0.05) in 3~5 *μ*m and unity emittance in 5~8 *μ*m. With those radiative properties, one can calculate the surface temperature of the structures (*T*_s_) through lumped capacitance method shown in Figure S1. This model is described in detail as follows.

Table s1 The presupposed spectral emittance of two structures

|  | 3~5 *μ*m | 5~8 *μ*m | Rest band |
| --- | --- | --- | --- |
| Surface 1 | 0.05 | 0.05 | 0.05 |
| Surface 2 | 0.05 | 1 | 0.05 |


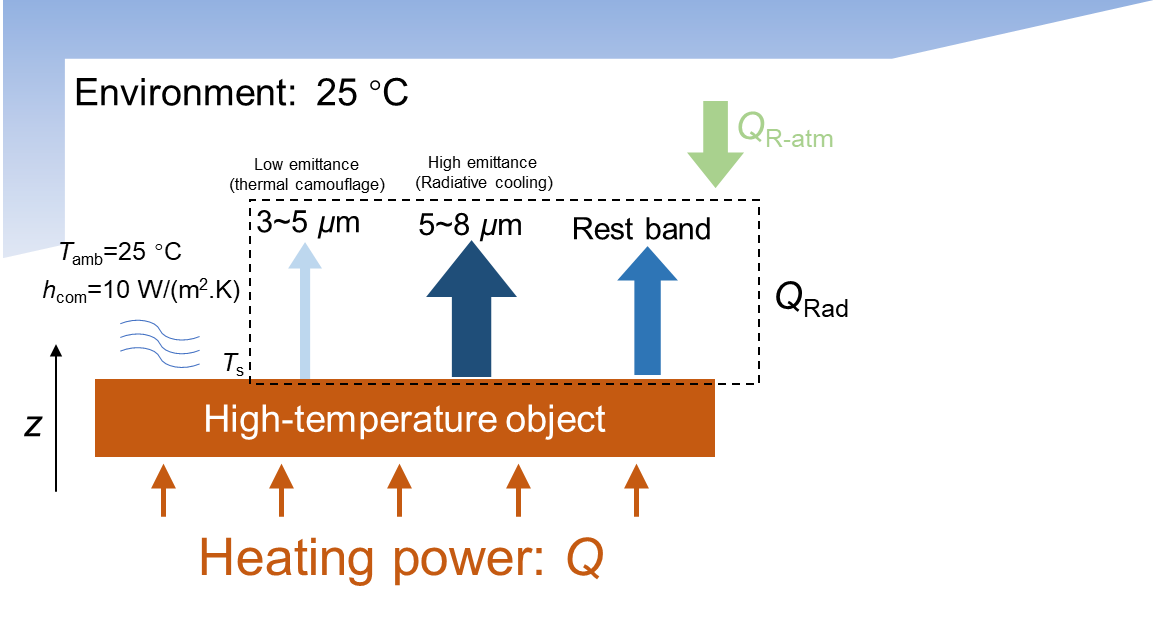


Figure s1 Scheme of thermal camouflage in 3~5 *μ*m and radiative cooling in 5~8 *μ*m of the high-temperature object with heat transfer process

As shown in Figure S1, a high-temperature object is heated under a power of *Q*. Under steady-state condition [1], this heating power can be expressed by

$Q=\text{ }Q_{\text{Rad}}\left(\varepsilon,T_{\text{s}}\right)+Q_{\text{com}}\left(h_{\text{com}},T_{\text{amb}},T_{\text{s}}\right)-Q_{\text{R-amb}}\left(\varepsilon,\varepsilon_{\mathrm{amb}},T_{\text{amb}}\right)$ (s1)

Here *T*_s_ and *T*_amb_ are the surface temperatures of the object and ambient. *Q*_rad_ signifies the thermal radiation emitted by the object (which is proportional to the fourth power of its temperature, multiplied by the Stefan-Boltzmann constant). *Q*_com_ represents the heat loss caused by conductive and convective heat exchange with the ambient, corresponding to *h*_com_ (the combined non-radiative heat transfer coefficient) as illustrated in Figure s1. *Q*_R-amb_ is the absorbed thermal radiation by the object from the ambient. The radiation power^[1]^ of the object can be expressed as

$Q_{\text{Rad}}\left(\varepsilon,T_{\text{s}}\right)=A\int\int I_{\text{BB}}\left(T_{\text{s}},\lambda\right)\varepsilon\left(\lambda,\theta\right)\text{d}\lambda\cos\theta\text{d}\Omega$ (s2)

where $\int\text{d}\Omega\text{=2π}\int_{0}^{\text{π}/2} \sin\theta\text{d}\theta$is the angular integral over a hemisphere. $I_{\text{BB}}\left(T_{\text{s}},\lambda\right)=\frac{2\text{hc}^{2}}{\lambda^{5}}\frac{1}{\text{e}^{\text{hc}/\left(\lambda\text{k}_{\text{B}}T\right)}-1}$ is the spectral radiance of a blackbody at temperature *T*_s_, where h is Planck’s constant, k_B_ is the Boltzmann constant, c is the speed of light in vacuum, *A* is the area. $\varepsilon\left(\lambda,\theta\right)$ is the wavelength and angular dependent emittance of the surface, which can be found in Table s1. *Q*_com_ is the heat loss of the object due to the combination of the convection and conduction heat transfer between the object and the ambient air, which can be calculated by

$Q_{\text{com}}\left(h_{\text{com}},T_{\text{amb}},T_{\text{s}}\right)=h_{\text{com}}A\left(T_{\text{s}}-T_{\text{amb}}\right)$ (s3)

with the air temperature (*T*_amb_) being 25 ℃ and *h*_com_ being the non-radiative heat transfer coefficient (assuming natural convection with *h*_com_=10 W/(m^2^∙K) in this work). The absorbed radiative heat of object from the environment can be calculated by

$Q_{\text{R-amb}}\left(\varepsilon,\varepsilon_{\mathrm{amb}},T_{\text{amb}}\right)=A\int\int I_{\text{BB}}\left(T_{\text{amb}},\lambda\right)\alpha\left(\lambda,\theta\right)\varepsilon_{\text{amb}}\left(\lambda,\theta\right)\text{d}\lambda\cos\theta\text{d}\Omega$ (s4)

where *T*_amb_ is the temperature of the environment (i.e., 25 ℃). $\alpha\left(\lambda,\theta\right)$ is the wavelength and angular dependent absorbance of the object, which equals $\varepsilon\left(\lambda,\theta\right)$ according to the Kirchoff’s law^[1]^ ($\alpha\left(\lambda,\theta\right)\text{=}\varepsilon\left(\lambda,\theta\right)$). For simplify in this work, the emittance of the ambient is assumed to be 1. According to the above calculations, the surface temperature of the object can be determined.

Note S2 The definition of IR signal intensity

The IR signal intensity of an object is estimated by integrating the IR radiant exitance in the concerned wavelength band (λ1~λ2) ^[2]^:

$$I=\int_{\lambda1}^{\lambda2} \varepsilon\left(\lambda\right)M_{\text{bb}}\left(\lambda,T\right)d\lambda$$

where *M*_bb_(λ,T) is the blackbody spectral radiant exitance at a given temperature *T* and *ε*(λ) is the surface emittance. The concerned wavelength bands (i.e., λ1~λ2) in this work are 3~5 *μ*m for thermal camouflage and 5~8 *μ*m for radiative cooling.

Note S3 The radiative temperature calculation

To obtain the radiative temperature of an opaque object, one can use an infrared camera. This infrared camera will receive the emitted radiation of the object (*P*_rad_) and the ambient radiation reflected by the object (ambient radiation reflection, *P*_ref_), by which the radiation temperature of this object is indicated. This radiative temperature, which is detectable by an infrared camera functioning in the 3~5 *μ*m range in this work, can be calculated using the inverse function of *P*(*ε*_i_, *T*):

$T_{\text{r}}=P^{-1}\left(\varepsilon_{\text{i}},T\right)$ (s5)

where *ε*_i_ is the default emittance (usually *ε*_i_ *=*1) in the infrared camera. *P*(*ε*, *T*) includes the emitted thermal radiation of the object (*P*_rad_) and the ambient radiation reflected by the object (*P*_ref_) with a detailed description, as follows:

$\begin{matrix} P\left(\varepsilon,T\right)=P_{\text{rad}}\left(\varepsilon,T_{\text{s}}\right)+P_{\text{ref}}\left(\varepsilon,\varepsilon_{\text{amb}},T_{\text{amb}}\right) \\ \begin{matrix} & & \end{matrix}=C\int_{3\mu\text{m}}^{5\mu\text{m}} \varepsilon\left(\lambda\right)I_{\text{BB}}\left(\lambda,T_{\text{s}}\right)d\lambda+C\int_{3\mu\text{m}}^{5\mu\text{m}} \left(1-\varepsilon\left(\lambda\right)\right)\varepsilon_{\text{amb}}I_{\text{BB}}\left(\lambda,T_{\text{amb}}\right)d\lambda\end{matrix}$ (s6)

where *ε*_amb_ is the spectral emittance and *C* is the angle integral constant (assuming *C* = 1) [2]. In this work, the ambient is 25 ℃ with its emittance *ε*_amb_≈1, as the surroundings can be regarded as a blackbody. According to the above calculations, the radiative temperature captured by the infrared camera can be determined.

Note S4 Transfer-Matrix Method (TMM)

The spectral properties of a multilayer structure can be calculated using the matrix formulation ^[3, 4]^. A multilayer structure containing *N* layers is shown in Figure s2. Each layer is assumed to be isotropic and homogeneous, and it can be described in terms of the relative permittivity *ε*l and relative permeability *μ*l (l=1, 2, . . . , *N*). The detailed calculation process to obtain reflectance and transmittance can be found in Ref [3, 4].


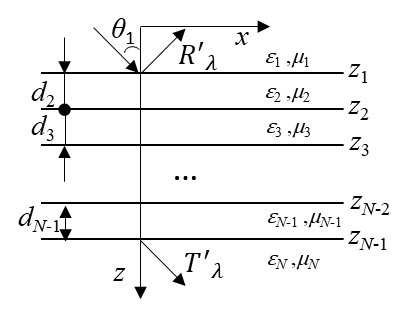


Figure s2 Schematic illustration of an *N*-layer structure, where the first and last layers are semi-infinite and each layer is assumed to be homogeneous and isotropic.

In practical applications, thin films are usually deposited on a thick substrate. Unlike the thin films, the light in a thick substrate can be considered incoherent. The transmittance and reflectance can be evaluated using the ray-tracing method.


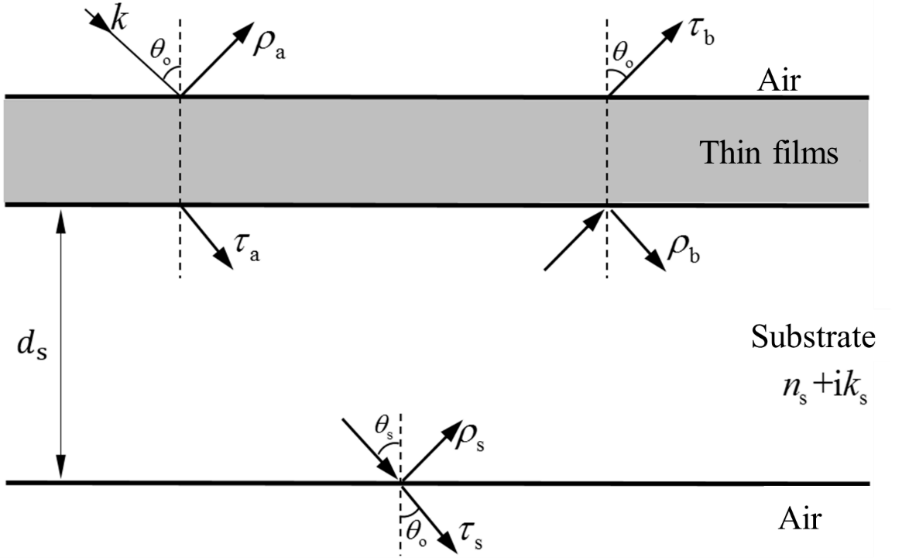


Figure s3 Radiative properties of multilayer thin films on an incoherent, thick substrate.

Figure s3 shows the geometry of a multilayer thin film on an incoherent substrate. It is assumed that the thickness of the substrate is *d*_s_, and the refractive index and the extinction coefficient of the substrate are $n_{s}$ and $k_{s}$, respectively. The refraction angle *θ*_s_ in the substrate can be calculated from the incidence angle *θ*_o_ by neglecting absorption of the substrate. In Figure s3, $\rho_{a}$ or $\rho_{b}$ refers to the reflectance of the first multilayer structure for rays originating from air or the substrate, respectively, and $\tau_{a}$ and $\tau_{b}$ are the corresponding transmittances. Furthermore, $\rho_{s}$ and $\tau_{s}$ represent the reflectance and transmittance of rays originating from the substrate at the interface of the substrate and air. They can be obtained from Fresnel’s formulas:

$\rho_{\text{s}}=\left(\frac{\eta_{\text{s}}-\eta_{0}}{\eta_{\text{s}}+\eta_{0}}\right)\left(\frac{\eta_{\text{s}}-\eta_{0}}{\eta_{\text{s}}+\eta_{0}}\right)^{\text{*}}$ (s21)

and $\tau_{\text{s}}=\left|\frac{4\eta_{\text{s}}\eta_{0}}{\left(\eta_{\text{s}}+\eta_{0}\right)^{2}}\right|$ (s22)

where $\eta_{s}$ and $\eta_{0}$ are the modified admittance of the substrate and air, respectively. For a TE wave, these values are $\eta_{s}=\left( n_{s}+ik_{s} \right)\cos\theta_{s}$ and $\eta_{0}=\cos\theta_{0}$, while for a TM wave, they are $\eta_{s}=\left( n_{s}+ik_{s} \right)/{\cos\theta_{s}}$ and $\eta_{0}=1/{\cos\theta_{0}}$. Because the coupling effect between the incident and reflected waves in the substrate is negligible, the transmittance is the same whether the ray originates from air or the substrate, i.e., $\tau_{a}=\tau_{b}$. The internal transmittance of the substrate is

$\tau\text{=}\exp\left(-\frac{4\pi k_{\text{s}}d_{\text{s}}}{\lambda\cos\theta_{\text{s}}}\right)$ (s23)

where λ is the wavelength in a vacuum. The reflectance and the transmittance of the multilayer structure can be calculated using the ray-tracing method and expressed as follows:

$R=\rho_{\text{a}}+\frac{\rho_{\text{s}}\tau_{\text{a}}^{2}\tau^{2}}{1-\rho_{\text{s}}\rho_{\text{b}}\tau^{2}}$ (s24)

and $T=\frac{\tau_{\text{s}}\tau_{\text{a}}\tau}{1-\rho_{\text{s}}\rho_{\text{b}}\tau^{2}}$ (s25)

The parameters $\rho_{a}$, $\tau_{a}$, $\rho_{b}$, and $\tau_{b}$ can be calculated using the matrix formulation introduced previously. According to the matrix formulation and the ray-tracing method, the radiative properties of arbitrary numbers of thin films on a thick substrate can be obtained. The applied substrates in this work is hard silica and soft 50-μm-thick PET. The refractive index of the materials applied in this work can be found in the database Ref ^[5]^.

Note S5 The description of Particle Swarm Optimization (PSO)

Particle Swarm Optimization (PSO) offers several advantages, making it a popular choice for solving optimization problems [6-12]. 1. Simplicity: PSO is conceptually simple and easy to implement. It uses a straightforward mechanism based on the movement and intelligence of swarms. 2. Few Parameters to Adjust: Unlike other optimization algorithms, PSO requires only a few parameters to be set, such as the number of particles, inertia weight, and acceleration coefficients. This reduces the complexity of the tuning process. 3. Robustness: PSO is robust and can handle a wide variety of optimization problems, including those that are nonlinear, non-differentiable, and multi-modal. 4. Fast Convergence: PSO can converge quickly to a good solution. It leverages the collective behavior of particles to explore and exploit the search space efficiently. 5. No Gradient Information Required: PSO does not require gradient information to find the optimum solution. This makes it suitable for problems where the objective function is not differentiable or the gradient is difficult to compute. 6. Scalability: PSO can be easily scaled to handle large and complex problems. It can be parallelized to improve computational efficiency. 7. Flexibility: PSO can be combined with other optimization techniques to enhance its performance. It can be adapted to different types of optimization problems by modifying the particle movement rules or incorporating domain-specific knowledge. 8. Global Search Capability: PSO maintains a population of potential solutions, allowing it to perform a global search of the solution space. This helps in avoiding local optima and finding the global optimum. Overall, PSO's simplicity, robustness, and efficiency make it a valuable tool for optimization in various fields, including engineering, computer science, and economics.

PSO is initialized with a population of random solutions (i.e., particles) ﬂown through a hyper dimensional search space ^[6, 7]^. The particles move in the search space by following the current optimum ones. One is the personal best solution that has been achieved so far, which is denoted as pbest. The other important parameter for PSO is the global best value obtained so far by any particle in the swarm, which is denoted as gbest. Supposing that the search space is d-dimensional, the *i*th particle of the swarm can be represented by a d-dimensional vector *Xi* = (*x_i_*_1_, *x_i_*_2_, ... , *x_i_*_d_)^T^. The velocity of this particle is represented by another d-dimensional vector *Vi* = (*v_i_*_1_, *v_i_*_2_, ... , *v_i_*_d_)^T^. The pbest of the *i*th particle can be denoted as P*i* = (*p_i_*_1_, *p_i_*_2_, ... , *p_i_*_d_)^T^ and the gbest can be denoted as G=(g_1_, g_2_, …, g_d_). After ﬁnding the pbest and gbest, the velocity of each particle for each dimension is updated with the following equation：

$v_{i\text{d}}^{t}=\omega\cdot v_{i\text{d}}^{t-1}+c_{1}\cdot r_{1}\cdot(p_{i\text{d}}^{t-1}-x_{i\text{d}}^{t-1})+c_{2}\cdot r_{2}\cdot\left(\mathrm{g}_{\text{d}}^{t\text{-1}}-x_{i\text{d}}^{t-1}\right)$ (s26)

where *ω* is a parameter known as the inertia weight representing the particle’s ﬂy without the inﬂuence of pbest and gbest. The parameter *r*_1_ and *r*_2_ are random numbers between (0, 1), and *c*_1_, *c*_2_ are the learning factors. The parameter *c*_1_ represents the inﬂuence of the particle’s memory from its best position, while the parameter *c*_2_ represents the inﬂuence of the global best position. The superscripts denote the iteration number. After each iteration, the position of each particle in the d-dimension is updated using the expression:

$x_{id}^{t}=x_{id}^{t-1}+\Delta t\cdot v_{id}^{t}$ (s27)

where $\Delta t$ is a given time step, usually equals 1.

In this paper, the defined objective function is the highest. The flowchart of the design process is shown in Figure s4. The thickness of each layer of the multi-layer structure is optimized by PSO relying on the objective function until reaching the iteration number. We set the thickness of each layer is thinner than 100 nm. After optimization, the gbest would be output. The program code is written in Matlab software. In a PSO algorithm, the parameters to be determined are: the swarm size (or population size), *c*_1_ and *c*_2_, the inertia weight *ω* and the maximum number of iterations. In order to enlarge the search space ^[13]^, a random number for inertia weight (*ω*) was set in the range [0.4, 0.9] and random numbers for the constants *c*_1_ and *c*_2_ were set in the range [1.49, 2.0]. The swarm size of particles was set 1000 and the number of iterations was set as 200 because the solutions would have almost no change for larger values.


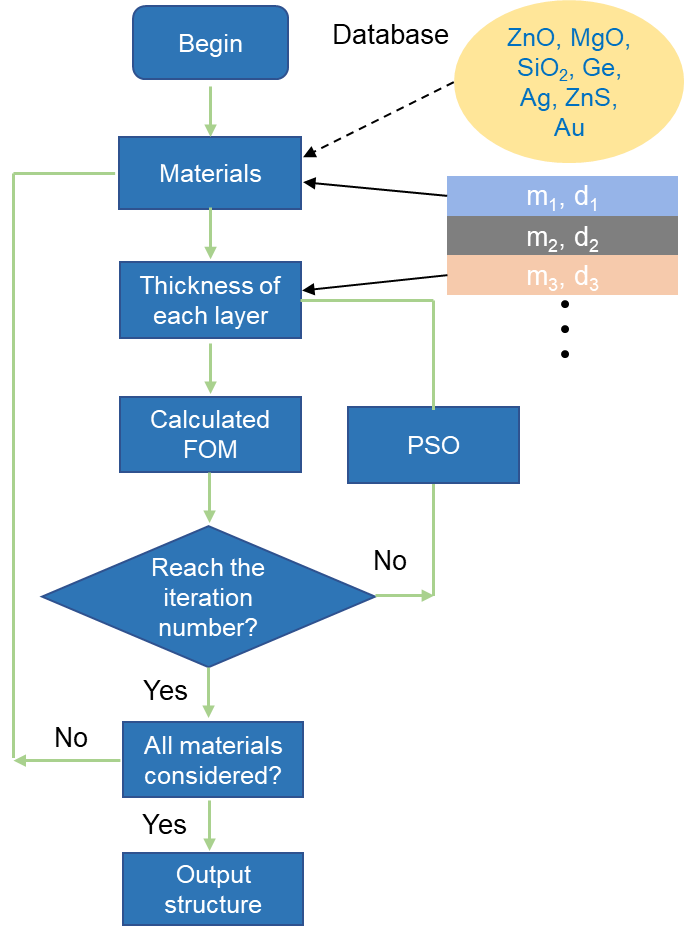


Figure s4 The flowchart of the design process of PSO.

Note S6 Infrared emittance measurement

To measure the direct-hemispherical infrared reflectance of the samples, a FTIR spectrometer (Bruker VERTEX 80) with a detector of DTGS an external golden integrating sphere (A562) was used, and the beam impinges on the sample with an incident angle of 13. To get the emittance of sample at room temperature, the reflectance is measured firstly, and the emittance can be calculated by 1-Reflectance. To obtain the emittance of a high-temperature object, The source of the high-temperature blackbody is SiC. By comparing the emitted thermal radiation from a blackbody (*S*_B_) and the structure (S_S_) at the same controlled temperature (detailed setup can be found in Figure s5), the emittance of the structure can be determined by

$\varepsilon=\frac{S_{\text{S}}-S_{\text{amb}}}{S_{\text{B}}-S_{\text{amb}}}$ (5)

S_amb_ represents ambient thermal radiation.

**
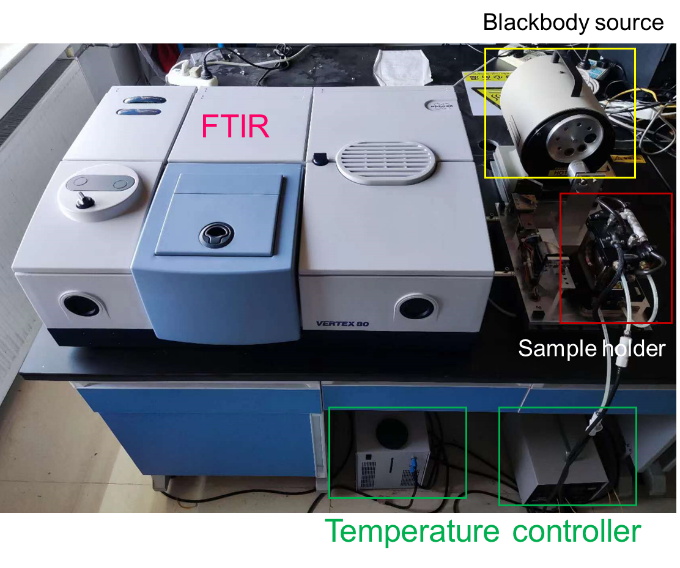
**

Figure s5 setup for high-temperature infrared emittance measurement.

**References**

[1] Bergman, T. L., Incropera, F. P., DeWitt, D. P., & Lavine, A. S. (2011). Fundamentals of heat and mass transfer. John Wiley & Sons.

[2] Zhu, H., Li, Q., Zheng, C., Hong, Y., Xu, Z., Wang, H., & Qiu, M. (2020). High-temperature infrared camouflage with efficient thermal management. Light: Science & Applications, 9(1), 60.

[3] Z. Zhang 2007 Nano/Microscale Heat Transfer. New York: McGraw-Hill.

[4] B. Zhao and Z. Zhang. Study of magnetic polaritons in deep gratings for thermal emission control. Journal of Quantitative Spectroscopy and Radiative Transfer 135 (2014) 81-89.

[5] Refractive index database: <https://refractiveindex.info/>

[6] Eberhart R, Kennedy J. A new optimizer using particle swarm theory, Micro Machine and Human Science, 1995, Proceedings of the Sixth International Symposium on. IEEE.

[7] Shi, Yuhui, and Russell C. Eberhart. Empirical study of particle swarm optimization. Evolutionary computation, 1999.. Proceedings of the 1999 congress on. Vol. 3. IEEE.

[8] Kennedy, J., & Eberhart, R. (1995). Particle swarm optimization. In Proceedings of ICNN'95 - International Conference on Neural Networks (Vol. 4, pp. 1942-1948). IEEE.

[9] Clerc, M., & Kennedy, J. (2002). The particle swarm-explosion, stability, and convergence in a multidimensional complex space. IEEE Transactions on Evolutionary Computation, 6(1), 58-73.

[10] Poli, R., Kennedy, J., & Blackwell, T. (2007). Particle swarm optimization. Swarm Intelligence, 1(1), 33-57.

[11] Van den Bergh, F., & Engelbrecht, A. P. (2004). A study of particle swarm optimization particle trajectories. Information Sciences, 176(8), 937-971.

[12] Engelbrecht, A. P. (2007). Computational intelligence: an introduction. John Wiley & Sons.

[13] S. K. Goudos and J. N. Sahalos, Microwave absorber optimal design using multi-objective particle swarm optimization, Microw. Opt. Technol. Lett., 48 (2006), pp. 1553-1558.
